# Insights into neurodegeneration from electron microscopy studies

**DOI:** 10.1042/BST20210719

**Published:** 2021-11-23

**Authors:** R. Anthony Crowther

**Affiliations:** MRC Laboratory of Molecular Biology, Francis Crick Avenue, Cambridge Biomedical Campus, Cambridge CB2 0QH, U.K.

**Keywords:** alpha-synuclein, electron microcopy, neurodegeneration, tau protein

## Abstract

Neurodegenerative diseases, such as Alzheimer's disease and Parkinson's disease, pose an increasingly severe burden for individuals and society in an ageing population. The causes and mechanisms of the diseases are poorly understood and as yet there are no effective treatments. Some of the molecular complexes involved in degeneration have been identified and electron microscopy has provided an essential tool in the investigations. The focus of this review is to show how electron microscopy has contributed historically to the understanding of disease and to summarize the most striking current advances. It does not seek to cover in detail the recent technical developments in microscopy, involving better microscopes, better electron detectors and more powerful image processing techniques, which have made possible the new insights. In many instances pathological filament assemblies are associated with brain cells that die in the disease, causing the observed symptoms such as dementia or movement disorders. Using electron microscopy it is now possible to go beyond morphological descriptions to produce atomic structures of many of the filaments. This information may help to understand the seeding and assembly of the filaments, with the aim of finding small molecule inhibitors that could potentially provide a form of treatment for the diseases.

## Introduction

Many neurodegenerative diseases are characterized by the presence in the brain of abnormal fibrous deposits. Two of the most common conditions are Alzheimer's disease (AD) and Parkinson's disease (PD). In AD the intracellular neurofibrillary tangles are made of microtubule-associated protein tau and the extracellular neuritic plaques contain amyloid-β (Aβ). In PD the deposits are known as Lewy bodies or Lewy neurites and they contain filaments made of α-synuclein. Besides these two relatively common diseases there are various other rarer diseases characterized by tau deposits, known collectively as tauopathies, or by α-synuclein deposits, known as synucleinopathies. Cells in which deposits form eventually die, leading to effects such as dementia or movement disorders, according to which part of the brain is affected. Most cases of these diseases are sporadic but some are familial and caused by a dominant mutation in the gene coding for the deposited protein. It is therefore believed that the seeding, assembly and abnormal deposition of the mutant protein represent a gain of toxic function and a driving force for the onset and development of the disease [1]. Thus it is of great importance to understand the details of the structure and assembly of the abnormal filaments forming the deposits. Electron microscopy provides an essential tool for such studies.

The principal filamentous component of AD tangles was first visualized with the electron microscope by Kidd in 1963 [2]. The specimen he used was an embedded and sectioned brain sample, fixed and positively contrasted with heavy metal [3]. The morphological information obtained was limited but he correctly identified the structure as consisting of two strands helically wound round one another. He aptly coined the name paired helical filament (PHF) to describe the structure. Since that time improvements in specimen preparation and huge developments in microscope and imaging technology have transformed what is possible, such that we now have atomic structures of many of the abnormal filaments. This review will cover some of the historical background and highlight recent results obtained by electron microscopy, which have greatly improved our understanding of these pathological structures.

## A little history

To go beyond the results of Kidd, it was necessary to extract filaments from an Alzheimer brain and prepare them for microscopy by negative rather than positive staining [4]. The filament is then embedded in stain and the morphology becomes much clearer, although still limited to mapping the outer surface of the structure. However, a number of features emerged. It was clear that the PHF indeed consisted of two strands of subunits, since in rare cases regions of the filament consisting of a single strand could be seen. Also in broken filaments the ends were always sharp, not frayed, and the length distribution of short fragments did not fall into discernable classes. This strongly suggested that each strand consisted of stacked subunits of very limited extent in the axial direction.

Electron microscopy played a key role in identifying the molecular nature of the PHF. An antibody raised against proteolytically treated PHFs was shown to both label a band on polyacrylamide gels of extracted protein and to decorate filaments by immuno-electron microscopy (immuno-EM) using gold-labelled second antibodies [5]. This firmly established a link between an extracted protein component and the core of the PHF. Protein sequencing and subsequent cloning showing that the protein band corresponded to a fragment of microtubule associated protein tau [6]. The microtubule binding repeat region of tau formed the core of the filament, while the N- and C-terminal regions of the protein formed a proteolytically labile fuzzy coat on the surface [7]. Antibodies with defined epitopes combined with immuno-EM of filaments are useful in determining which parts of the tau sequence are buried and which exposed [8]. Tangles consist predominantly of PHFs but also contain morphologically distinct straight filaments (SFs) as a minor component. Computed cross-sectional maps showed that PHFs and SFs are formed from strands of the same stacked C-shaped unit but differently arranged [9]. Electron diffraction of individual tau filaments extracted from AD brain proved that the structure was predominantly of cross-β type, consistent with being designated an amyloid [10].

When studying different diseases, immuno-EM is useful in very impure brain extracts to determine which filaments are made of tau. It thus became clear that, in clinically distinct diseases involving tau protein, tau filaments displayed different morphologies [11]. In a transgenic mouse model of tauopathy to study induction and spreading of pathology after injection of brain extracts, immuno-EM proved that filaments were being newly assembled, not merely spreading from the site of injection [12].

Following the discovery of a point mutation in the α-synuclein gene in a rare familial case of Parkinson's disease (PD) [13], it was shown that α-synuclein was present in Lewy bodies in PD and in dementia with Lewy bodies (DLB) [14]. Filaments extracted from brains of patients with DLB [15], multiple system atrophy (MSA) [16] and PD [17] all showed strong decoration with anti-α-synuclein antibodies using immuno-EM, substantiating the grouping of these diseases as synucleinopathies. As with tau filaments in tauopathies, synuclein filaments from clinically different diseases had various morphologies. Electron diffraction from *in vitro* assembled α-synuclein filaments demonstrated that, like tau filaments, they had a cross-β structure [18].

This brief summary demonstrates the importance that electron microscopy as a general tool has had historically in studying neurodegenerative diseases. All the previous examples involving imaging used stained specimens, thus limiting the degree of detail obtained to general morphology. Specimens are now prepared by rapid freezing, so that the biological material itself is imaged without stain [19]. Also, recent advances in instrumentation and image processing have had a profound impact, so that for many of the samples mentioned atomic structures are now available. This in turn provides a much greater understanding of neurodegenerative diseases.

## Recent developments in electron microscopy

The recent advances in microscopy have depended on various technical developments. Microscopes have been improved with bright coherent electron sources and direct electron directors that record images with a quality limited mainly by electron statistics [20]. Powerful computational approaches based on Bayesian principles [21] allow extraction of the maximum reliable information from the images to determine three dimensional maps of the specimen. Frozen specimens embedded in amorphous ice are very radiation sensitive. Imaging is thus restricted to low electron doses before the specimen is damaged. The images are therefore very noisy and many have to be averaged together to extract detailed information about the specimen. The earliest maps interpretable in terms of atomic structure were of isolated molecules or complexes [22], giving rise to the term single particle cryo-EM. More detailed accounts of advances in cryo-EM can be found in references [23,24].

Of particular relevance to neurodegenerative disease, amongst early atomic structures determined by single particle methods, was that of the human γ-secretase [25]. This intra-membrane protease is the enzyme responsible for one of the proteolytic cleavages that creates Aβ fragments from the amyloid precursor protein (APP), itself a membrane protein. γ-secretase is a complex of four integral membrane subunits with a total protein mass of ∼170 kDa. Most of the mutations that give rise to familial AD map to the presenilin subunit or to APP itself, suggesting an important role for Aβ creation and deposition in the development of the disease. The γ-secretase is highly glycosylated and was stabilized after extraction in amphipols, making it an unsuitable target for crystallization. These factors proved not to be a problem for single particle cryo-EM, which yielded an atomic structure for most of the complex, though parts of the catalytic subunit were disordered [25]. These structural variations were actually exploited by further sophisticated developments of image classification to identify three distinct conformations of the catalytic subunit in the population of molecules present, giving information about the mechanism of cleavage [26]. This demonstrates the power of cryo-EM to analyze a heterogeneous population, whether in conformation or occupancy, of molecules or complexes to produce detailed three dimensional maps of distinct conformers or components, the separation being done *in silico* from images of the mixed population. If different conformations are detected, they may then give information about the function or mechanism of the complex.

As mentioned, many structures of interest in neurodegenerative diseases are filamentous, so single particle methods of the kind just described are not immediately applicable. However, an extended filament can be turned into single particles by dividing the image into a set of short overlapping segments. Filaments have helical symmetry but the short-range order is usually much better than the long-range order. Division of the image into segments overcomes this problem, as each short segment is well ordered and determination of the view parameters of each segment corrects for the long-range disorder. However, the predominantly cross-β structure of amyloid filaments, with a 4.7 Å spacing of β-strands along the helical axis but with little larger scale modulation in shape along the filament, means that reliable determination of orientation of the segments can be difficult. Special adaptations of single particle methods have therefore been made to allow the underlying helical symmetry to contribute optimally to the structure determination [27,28]. Successful application of these methods means that atomic structures of many pathological filaments have now been determined and some of these results will now be described.

## Tau filaments

As already mentioned, microtubule-associated protein tau forms filaments in various neurodegenerative diseases, including AD, where it forms PHFs and SFs. In adult human brain there are six isoforms of tau produced by alternative mRNA splicing [29]. A repeat region of 31 or 32 amino acids in the C-terminal half of the protein forms the microtubule binding region of tau and three isoforms have 3 repeats (3R) and three isoforms have 4 repeats (4R), with repeat 2 (R2) being present in 4R but not 3R isoforms. In addition, in the N-terminal part of tau there can be 0, 1 or 2 inserts forming the six isoforms 0N3R, 1N3R, 2N3R, 0N4R, 1N4R, and 2N4R. The longest isoform, which forms the basis for the amino acid numbering scheme, has 441 amino acids. Early work on AD showed that part of the repeat region formed the core of PHFs [5] and that all 6 isoforms were included in the filaments [8]. In chronic traumatic encephalopathy (CTE), a disease associated with brain trauma, the filaments also contain all 6 isoforms but in Pick's disease (PiD) filaments contain only 3R isoforms, while in corticobasal degeneration (CBD) they are made from 4R isoforms [30]. Atomic structures determined by cryo-EM help to explain these differences.

**Figure 1.**
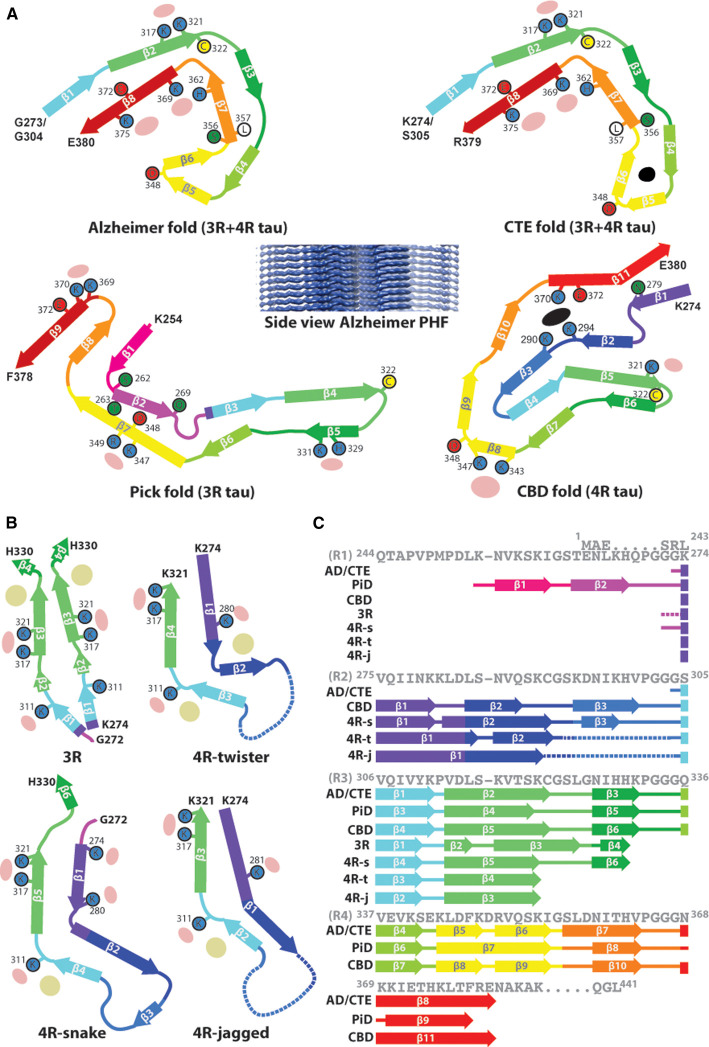
Structures of tau filaments. (**a**) Folds of tau protein in the cores of filaments from various diseases: Alzheimer's disease, chronic traumatic encephalopathy, Pick's disease and corticobasal degeneration. A side view of the Alzheimer PHF is also shown. (**b**) Folds observed in heparin assembled filaments do not match any of the disease folds. (**c**) Sequence of the repeat region of tau, showing the part of the sequence in each core filament and the segments corresponding to the β-strands. Reproduced with permission from Scheres et al. [36].

The first filaments to be solved were PHFs and SFs from AD patients [31]. The two types of filament showed a common C-shaped AD fold (Figure 1a) but with the two strands of subunits differently related. Amino acids 273/304–380 formed the ordered core with the remainder of the protein disordered on the outside of the filament. The fold was formed by β-strands lying roughly in the plane normal to the helical axis, with the stacking of subunits at an axial spacing of 4.7 Å forming the cross-β structure expected (see side view in Figure 1a). The fold could accommodate both 3R and 4R isoforms, with sequences from repeats R3 and R4 forming the majority of the structure. Weak additional densities of unknown origin are associated with lysine residues on the surface of the filaments. The structure of filaments from multiple cases, both sporadic and familial, and from different brain regions was identical [32], confirming that the AD fold is characteristic of the disease. The fold in CTE filaments (Figure 1a) is similar to the AD fold, with the C-shape of the subunit slightly opened up but still accommodating 3R and 4R isoforms [33]. In the CTE map there is extra density occupying a hydrophobic pocket in the tip of the subunit, implying the presence of non-proteinaceous material inside the filament.

The fold in the Pick filament (Figure 1a) is very different from the AD or CTE fold, with a larger ordered part corresponding to amino acids 254–378 from repeats R1, R3 and R4 [34]. Importantly, the Pick fold cannot accommodate repeat R2 because of differences in sequence between R1 and R2, so Pick filaments can only contain 3R isoforms, as observed. As a final example of tauopathy, the fold in filaments from CBD (Figure 1a) is different again, comprising amino acids 274–380 and incorporating sequences from repeats R2, R3 and R4 [35]. It is thus clear that, as was already known, the CBD fold can only be formed by 4R isoforms, since 3R isoforms do not contain repeat R2. As with the CTE fold, there is additional density in an internal pocket, but in CBD the pocket is lined by charged rather than hydrophobic residues. In the tau filaments in the various diseases most of the β-stranded regions are in common (Figure 1c) but folded differently by alternative conformations at the bends joining the β-strands. More detailed comparisons of these tau filament structures are given in [36]. Recently determined structures of filaments from various other diseases have led to a powerful structure based classification scheme for tauopathies, based on the number of layers in the β-fold [37].

Assembly of tau filaments *in vitro* using heparin [38] has been much used but detailed atomic structures of the resulting filaments (Figure 1b) have now shown that none of the forms observed recapitulates the fold seen in AD or the other tauopathies [39]. It is thus essential, when studying tau filaments, to use filaments isolated from brain to establish the relevant pathological structures. These can then be used to validate approaches to forming filaments *in vitro* for studies of assembly mechanisms or possible blocking strategies.

Detailed atomic analysis of filaments depends on post-mortem material. However it is also important to be able to detect tau filaments in living subjects and one way of doing this is to use positron emission tomography (PET), with ligands specific for assembled tau. To understand the specificity it is important to know how the small molecule ligands interact with the filaments. Cryo-EM has been used to determine the binding sites to AD filaments of one such ligand APN-1607 [40]. Comparing maps of filaments with and without ligand gives a series of difference peaks that can be interpreted as the binding of APN-1607 parallel to the helix axis, in sites within the C-shaped cavity of the AD fold. However because the APN-1607 molecule is about five times longer than the 4.7 Å cross-β spacing, it is not in register with that spacing and the difference peaks cannot be interpreted in atomic detail. Nevertheless, the study demonstrates the potential for cryo-EM to determine the positions of small molecules bound on filaments and gives useful information about the mode of binding. This may be exploited to design better labels that discriminate between different tau filaments and thus aid the diagnosis of different diseases.

## Other filaments

**Figure 2.**
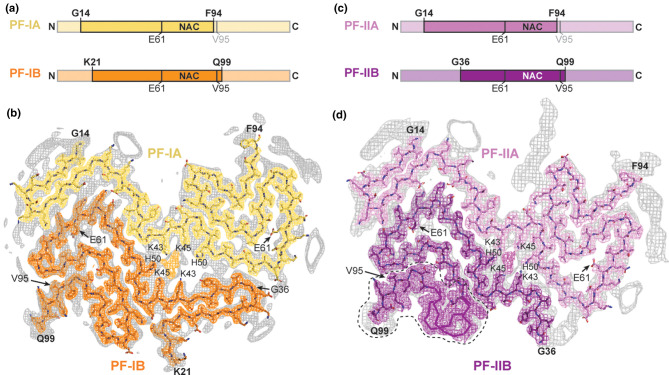
Structures of the two types of α-synuclein filament seen in MSA. (**a**) The two segments of sequence found in the two different protofilaments of type I filaments. (**b**) Cryo-EM map and superimposed model of the fold in the type I filament. (**c**) The two segments of sequence found in the two different protofilaments of type II filaments. (**d**) Cryo-EM map and superimposed model of the fold in the type II filament. Reproduced with permission from Schweighauser et al. [41].

Synucleinopathies represent another widespread class of neurodegenerative diseases involving abnormal filament formation, in this case by α-synuclein. Of these, Parkinson's disease is the most prevalent but it has not so far proved possible to obtain an atomic structure of PD filaments or of DLB filaments. However the filaments from brain in MSA cases have been solved in atomic detail [41]. Like tau, α-synuclein is a natively unfolded protein and it contains 140 amino acids. The MSA study showed that there are two kinds of filament, termed type I or type II, each consisting of two protofilaments. But unlike PHFs and SFs in AD, in which the tau fold is identical in the two protofilaments, in MSA the α-synuclein fold is different in the two protofilaments (Figure 2). As with tau filaments, the fold of the protein lies predominantly in a plane normal to the helical axis and the cross-β structure is formed by the stacking of these planar segments. Again as with tau filaments, the core of the filament is made from an internal segment of the protein with the N- and C- terminal regions disordered on the outside. In the interface between the two protofilaments in both type I and type II filaments there is a strong additional peak of density that does not correspond to part of the protein. Thus, as with tau filaments in CTE and CBD, there is a cofactor of some kind involved in their assembly.

There have been multiple studies using cryo-EM of *in vitro* assembled α-synuclein filaments (e.g. [42,43,44]). Although there are similarities in the fold, the MSA filaments from brain have an extended ordered core region compared with the *in vitro* ones. As mentioned, they also exhibit asymmetry between the two protofilaments, whereas the *in vitro* ones are symmetric. Furthermore, the brain derived filaments contain additional material not seen in the *in vitro* ones. Even with seeded assembly of filaments driven by MSA seeds from brain, the structure of the resulting filaments does not match those from brain [45]. Therefore, as with tau filaments, more work is needed to produce *in vitro* systems that replicate the structures relevant to disease. Cryo-EM will be essential for monitoring the results of assembly to compare with authentic structures of brain derived filaments as targets.

Another technique that may have application for studying neurodegeneration is cryo-electron tomography (cryo-ET). Unlike imaging of dispersed single particles or filaments, cryo-ET can be used to study sections of frozen tissue or bundles of filaments. Multiple images of a specimen volume are collected by tilting the sample in the microscope, from which a three dimensional map of the material in the volume can be calculated. In models of Huntington's disease fibrous aggregates containing polyglutamine have been studied *in situ* in relation to other sub-cellular components [46] and details of filament structure in bundles elucidated [47]. Cryo-ET has also been used in work on α-synuclein inclusions within cultured neuronal cells, showing α-synuclein fibrils intermixed with membraneous organelles [48]. Individual tomograms have limited resolution but, if repeated features can be identified and extracted, sub-tomogram averaging allows more detailed features to be visualized [49].

**Figure 3.**
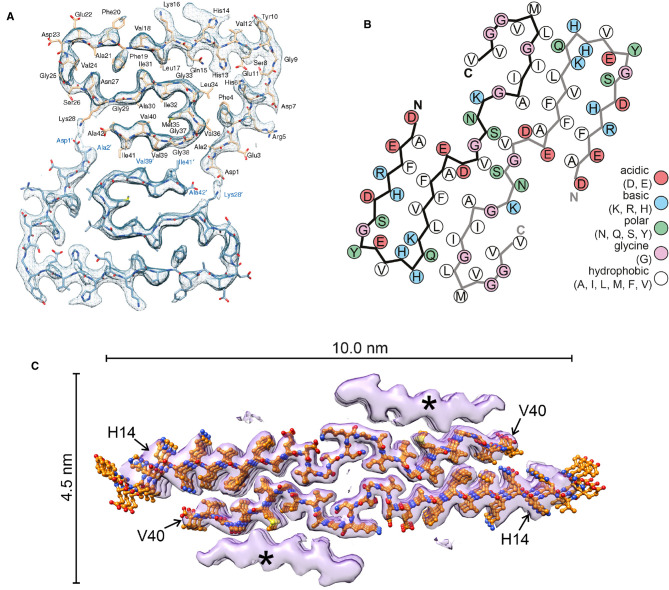
Folds of various Aβ amyloid fibrils. (**a**) In vitro assembled Aβ (42) [50]; (**b**) Aβ (40) filaments isolated from brain meninges [51]; (**c**) Assembled Aβ (40) seeded from brain material [52]. Reproduced with permission from references [50,51,52].

The Aβ peptide, which forms fibrous deposits in neuritic plaques in AD, occurs with different lengths between 40 and 43 amino acids, with the most abundant being Aβ (40) and Aβ (42). The different species have different aggregation and functional properties and the filaments formed from them are polymorphic. It has proved difficult to isolate Aβ filaments from brain, so many studies have been made with *in vitro* assembled filaments using peptides of different lengths, producing conflicting ideas about the structure of the filaments. Recent cryo-EM studies of *in vitro* assembled Aβ (42) [50], Aβ (40) filaments isolated from brain meninges [51] and Aβ (40) filaments seeded from brain material [52] have shown radically different folds for the peptide (Figure 3). Since the filaments contain different Aβ peptides and were assembled or isolated in different ways, it is perhaps not surprising that they yield different structures, as has been observed for tau and synuclein filaments. Clearly more work is needed to establish the relevant structure(s) of Aβ filaments in the brain, particularly those in neuritic plaques in AD, and to understand the differences in properties of the Aβ peptides of different length.

As a final example, the nucleic acid binding protein TDP-43 forms pathogenic filamentous aggregates in amyotrophic lateral sclerosis and other neurodegenerative diseases. The atomic structures of filaments made *in vitro* from amyloidogenic fragments of TDP-43 have been solved by cryo-EM, showing the existence of multiple polymorphs [53]. Filaments made from a much larger region of the protein have also been solved [54]. As with the other filaments already described, the relevance of these findings will have to be checked by reference to brain-derived samples.

## Summary

The examples given illustrate the power of electron microscopy for studying neurodegenerative diseases. A particularly important feature is the ability to analyse the frequently heterogeneous samples, whether single particles or filaments, using image classification. This can reveal different structures or conformations in a single set of micrographs. For filaments the results have shown that a given protein can present different folds in different diseases but in any one disease the folds are consistent. All the filaments solved to date have a common amyloid structure with a segment of the constituent protein forming a roughly planar β-strand core fold normal to the helical axis of the filament, with the termini of the protein disordered on the outside. A given protein can produce multiple folds from common β-strand regions linked by flexible joints. Maps of filaments isolated from brain show additional non-proteinaceous material, yet to be identified, which might explain why *in vitro* assembly lacking these cofactors has yet to reproduce filaments like those in brain.

## Perspectives

- In an ageing population neurodegenerative diseases impose a huge burden on individuals and society. Multiple approaches are needed to understand these complex diseases and electron microscopy provides the best method to determine the structures of relevant molecular complexes.
- We now have atomic structures of the abnormal filaments believed to drive pathology in various diseases. The natively unfolded proteins that assemble to form filaments fold in different ways to produce filaments characteristic of the different diseases.
- This information needs to be exploited to understand the mechanisms of seeding and assembly of the filaments. The non-proteinaceous components must be identified and reliable *in vitro* assembly protocols established, so small molecule inhibitors can be found and assessed for effectiveness.

## Open Access Statement

Open access for this article was enabled by the participation of MRC Laboratory of Molecular Biology in an all-inclusive Read & Publish pilot with Portland Press and the Biochemical Society under a transformative agreement with JISC.

## Abbreviations

AD: Alzheimer's disease
Aβ: amyloid-β
CBD: corticobasal degeneration
cryo-EM: cryo-electron microscopy
cryo-ET: cryo-electron tomography
CTE: chronic traumatic encephalopathy
DLB: dementia with Lewy bodies
immuno-EM: immuno-electron microscopy
MSA: multiple system atrophy
PD: Parkinson's disease
PET: positron emission tomography
PHF: paired helical filament
PiD: Pick's disease
SF: straight filament
